# P-1204. Gepotidacin activity against Escherichia coli and Klebsiella pneumoniae, including molecularly characterized fluoroquinolone not susceptible subsets causing urinary tract infections in United States Medical Centers (2023)

**DOI:** 10.1093/ofid/ofaf695.1397

**Published:** 2026-01-11

**Authors:** Rodrigo E Mendes, Zachary Kockler, Krisztina M Papp-Wallace, Renuka Kapoor, Nicole E Scangarella-Oman, S J Ryan Arends

**Affiliations:** Element Iowa City (JMI Laboratories), North Liberty, IA; Element Iowa City (JMI Laboratories), North Liberty, IA; Element-Iowa City, formerly JMI Laboratories, North Liberty, Iowa; GSK, Atlanta, Georgia; GlaxoSmithKline plc., Collegeville, PA; Element Iowa City (JMI Laboratories), North Liberty, IA

## Abstract

**Background:**

Gepotidacin (GEP) is a novel, bactericidal, first-in-class triazaacenaphthylene antibiotic that inhibits bacterial DNA replication by a distinct binding site, a unique mechanism of action and provides well-balanced inhibition of two type II topoisomerases (for most pathogens). GEP was recently approved by the United States (US) Food and Drug Administration (FDA) for the treatment of uncomplicated urinary tract infections (uUTI). This study reports the activity of GEP and other oral agents against *E. coli* (EC) and *K. pneumoniae* (KPN), including characterized fluoroquinolone (FQ) not susceptible (NS) isolates.

**Figure:**
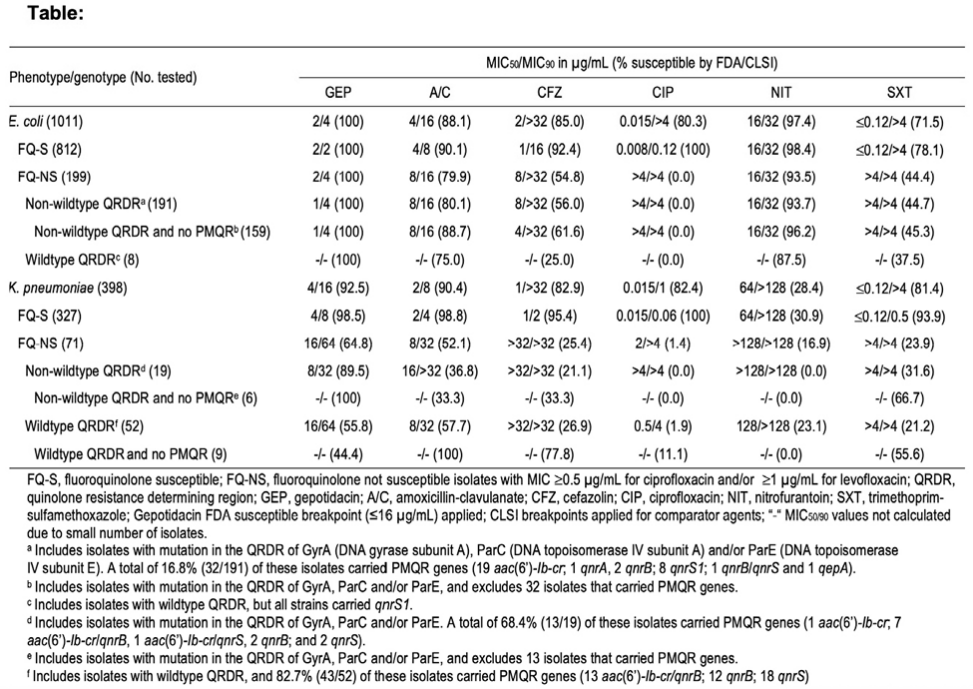

**Methods:**

1,409 isolates (1,011 EC and 398 KPN) from 58 US sites were included. CLSI methods were used for both susceptibility testing and MIC interpretation, except for GEP that used FDA breakpoints. Isolates that were NS to ciprofloxacin and/or levofloxacin were screened for FQ resistance (R) mechanisms (QRDR mutations, plasmid-mediated genes [PMQR]).

**Results:**

GEP had MIC_50/90_ values of 2/4 µg/mL against all EC, and inhibited all (100%) isolates at the FDA S clinical breakpoint of ≤16 µg/mL. Among comparators, only nitrofurantoin was active against more than 90% of EC. A total of 19.7% of EC were FQ-NS. GEP had MIC_90_ values of 4 µg/mL against FQ-NS EC, regardless of FQ R mechanisms, and all FQ-NS EC were S to GEP. GEP (MIC_50/90_, 4/16 µg/mL) was active against 92.5% of all KPN, while amoxicillin-clavulanate (MIC_50/90_, 2/8 µg/mL) showed the highest S result (90.4%) among comparators. Among KPN isolates, 17.8% were FQ-NS, and GEP (MIC_50/90_, 16/64 µg/mL) inhibited 64.8% of these isolates at the S breakpoint. At the S breakpoint, GEP inhibited 89.5% to 100% of FQ-NS KPN isolates with QRDR mutations, regardless of the presence of PMQR. GEP had MIC_50/90_ values of 16/64 µg/mL against FQ-NS KPN with wildtype QRDR, including those that carried PMQR.

**Conclusion:**

GEP demonstrated activity, with 100% susceptibility, against both FQ-S and FQ-NS EC causing UTIs in US medical centers, regardless of FQ R mechanisms. GEP was highly active against FQ-S KPN, and showed activity higher than comparator agents against FQ-NS KPN. These data benchmark GEP against EC and KPN for subsequent monitoring following its recent FDA approval for the treatment of uUTIs.

**Disclosures:**

Rodrigo E. Mendes, PhD, GSK: Grant/Research Support|Shionogi & Co., Ltd.: Grant/Research Support|United States Food and Drug Administration: FDA Contract Number: 75F40123C00140 Renuka Kapoor, PhD, GSK: Employee|GSK: Stocks/Bonds (Public Company) Nicole E. Scangarella-Oman, MS, GSK: Employee|GSK: Stocks/Bonds (Public Company)

